# Observation of a new superfluid phase for ^3^He embedded in nematically ordered aerogel

**DOI:** 10.1038/ncomms12975

**Published:** 2016-09-27

**Authors:** N. Zhelev, M. Reichl, T. S. Abhilash, E. N. Smith, K. X. Nguyen, E. J. Mueller, J. M. Parpia

**Affiliations:** 1 Department of Physics and Laboratory of Atomic and Solid State Physics, Cornell University, Ithaca, New York 14853, USA; 2 School of Applied and Engineering Physics, Cornell University, Ithaca, New York 14853, USA

## Abstract

In bulk superfluid ^3^He at zero magnetic field, two phases emerge with the B-phase stable everywhere except at high pressures and temperatures, where the A-phase is favoured. Aerogels with nanostructure smaller than the superfluid coherence length are the only means to introduce disorder into the superfluid. Here we use a torsion pendulum to study ^3^He confined in an extremely anisotropic, nematically ordered aerogel consisting of ∼10 nm-thick alumina strands, spaced by ∼100 nm, and aligned parallel to the pendulum axis. Kinks in the development of the superfluid fraction (at various pressures) as the temperature is varied correspond to phase transitions. Two such transitions are seen in the superfluid state, and we identify the superfluid phase closest to *T*_c_ at low pressure as the polar state, a phase that is not seen in bulk ^3^He.

## Abstract (web summary)

Anisotropic disorder is predicted to change the stability of the superfluid state in ^3^He and leads to new phases. Here, Zhelev *et al*. report signatures of the phase transitions in ^3^He confined in an extremely anisotropic ordered aerogel including a new polar phase.

## Graphical abstract

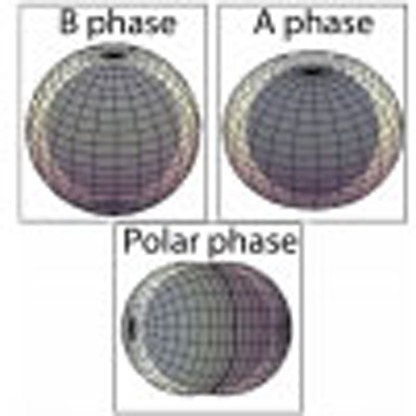

Superfluid ^3^He is our best example of a quantum system, where the fermionic constituents form Cooper pairs with finite angular momentum. Similar to unconventional superconductors, such as Sr_2_RuO_4_ and UPt_3_ (refs 1, 2), the properties of this exotic superfluid can be engineered with disorder^3^,^4^. Theory predicts that nanoscale confinement and anisotropic disorder profoundly change the stability of the pairing, and can lead to novel phases^5^,^6^,^7^,^8^.

Two superfluid phases—the A and B phases are observed in bulk ^3^He when it is cooled to ultralow temperatures (below 0.902–2.444 mK depending on the pressure)^9^. At these temperatures, bulk ^3^He is an extremely pure system free of any disorder: anything besides helium is solid and condenses on the surfaces, and ^4^He phase separates with practically zero solubility in the ^3^He. To deliberately introduce disorder, one can embed the fluid in highly porous aerogels^10^,^11^. For ^3^He in aerogel, *T*_c_ is suppressed and the relative stability of the A and B phases is markedly different compared with the bulk. The phase diagram is further distorted when weak anisotropy is introduced by compressing or stretching the aerogel^3^,^12^,^13^,^14^. In the experiment described here, we embed the fluid within a highly oriented nematic aerogel^15^,^16^,^17^, which provides extreme anisotropy. Nuclear magnetic resonance (NMR) experiments^15^,^16^,^17^ identify the resulting phases as polar-distorted A and B phases. Evidence for a novel polar phase was recently observed in a NMR measurement of a different high-density nematic aerogel^18^. In our experiments, the aerogel is mounted on a torsion pendulum and the strands that comprise the aerogel are aligned parallel to the pendulum axis. This technique is very different from NMR and is specifically sensitive to the in-plane superfluid fraction. Spin diffusion measurements in an aerogel sample similar to the one we study reveal that the mean-free path for a particle travelling along the strands is nearly twice that of one moving perpendicular^19^. From the period of this pendulum, we measure the fraction of the fluid that is decoupled from the container, which is related to the superfluid fraction. As we vary the temperature and pressure, we observe kinks when superfluid fraction is plotted versus temperature, corresponding to phase transitions. We find a new superfluid phase just below the normal to superfluid transition that is not seen in bulk ^3^He. Drawing on theoretical work^4^,^5^,^20^,^21^, we argue that at low pressure this new superfluid phase is the polar phase.

## Results

### Microstructure of the nematic aerogel

**Figure 1:**
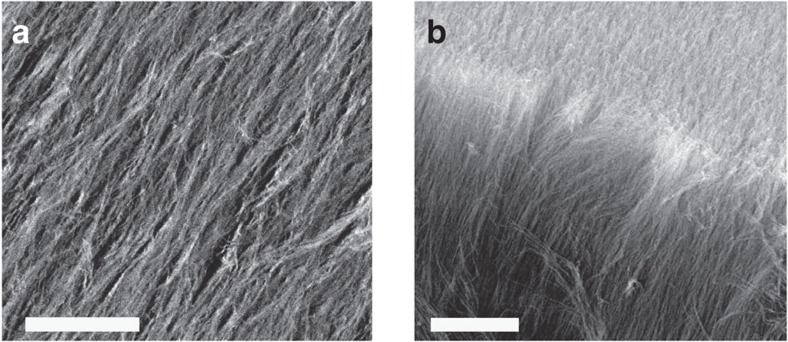
Scanning electron microscope (SEM) micrographs showing the microstructure of the aerogel sample used in this experiment. (**a**) SEM image in a plane parallel to the aerogel strands. Scale bar, 1 μm. (**b**) SEM image of the edge of the aerogel sample at the intersection of planes parallel and perpendicular to the strands. Scale bar, 5 μm. Images are taken at Cornell, using Tescan Mira3 Field Emission SEM.

Scanning electron microscope images of the aerogel sample showing its highly oriented microstructure are shown in Fig. 1a,b. We estimate that the aerogel consists of ∼10 nm-thick strands, spaced ∼100 nm apart.

### Ginzburg–Landau model

**Figure 2:**
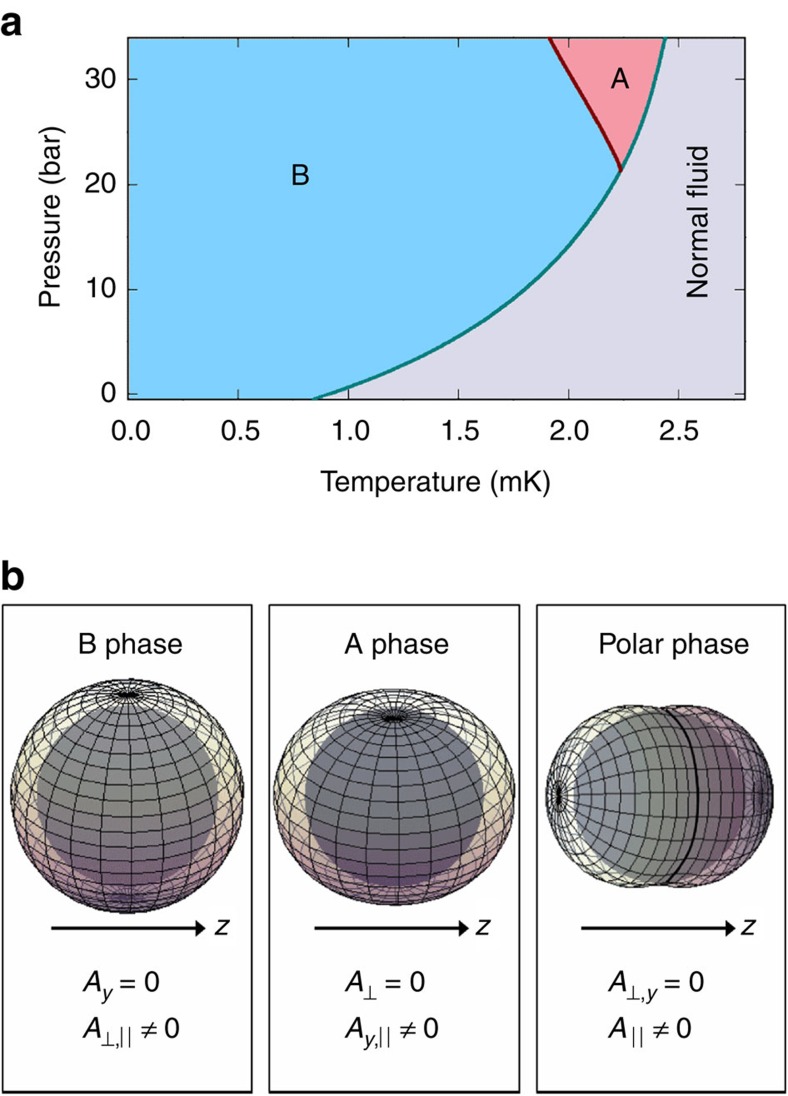
Bulk fluid phase diagram and visual representation of the superfluid order parameter. (**a**) Superfluid phase diagram for bulk ^3^He at zero magnetic field. The anisotropic superfluid A phase is realized only in a relatively small region at high pressures and at temperatures near the superfluid transition temperature. (**b**) Visual representation of the superfluid gap around the Fermi surface for various superfluid phases of ^3^He. The direction of largest superfluid gap is aligned with the aerogel strands along the *z* axis.

The order parameter for ^3^He is a rank-2 tensor describing the spin and orbital angular momentum of the pairs. In the bulk A-phase, the orbital angular momentum points in a fixed direction, and there exist gapless excitations whose momenta are parallel to this direction. In the B-phase, the superfluid gap is isotropic and nonzero in all directions around the Fermi surface. The polar phase, which is not stable in bulk ^3^He, has a nodal plane: for any momentum direction in this plane, one can find gapless quasiparticles. Larger superfluid densities are associated with having fewer low-energy excitations, allowing us to distinguish these phases. Figure 2a shows the bulk phase diagram, and Fig. 2b gives a visual representation of the superfluid energy gaps in the various phases.

Following convention, we represent the order parameter as a 3 × 3 matrix **A** with components related to the coupling of the *x*, *y* and *z* components of the spin and orbital angular momentum. All three phases that we see are captured by the ansatz 
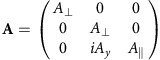
. The conventional bulk A-phase corresponds to *A*_*y*_=*A*_||_ and *A*_⊥_=0. Similarly, the isotropic B-phase has *A*_⊥_=*A*_||_ and *A*_*y*_*=*0. The polar phase has *A*_⊥_=*A*_*y*_=0. Guided by the form of the Ginsburg–Landau (GL) free energy in refs 4, 20, we write the free energy for this system including the fourth-order terms scaled by the appropriate coefficients *β*_*i*_ as follows:


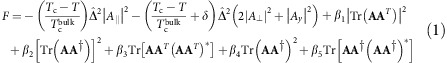


Here 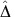 is the value of the condensation energy, and *T*_c_ and *T*_c_^bulk^ the superfluid transition temperatures for the fluid within the aerogel and the bulk fluid, respectively. The anisotropy in the system is parameterized by *δ*. The nonzero value of *δ* leads to two distinct transition temperatures, *T*_c⊥_ and *T*_c||_=*T*_c_, below which the components of the order parameter perpendicular and parallel to the aerogel anisotropy axis develop. The effect of the anisotropy of the system splitting the superfluid transition has been observed for Sr_2_RuO_4_ under uniaxial strain^22^. More precisely *δ* can be defined as:


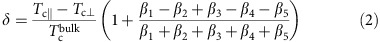


In the linear pair-breaking regime, *δ* is given by ref. 4:


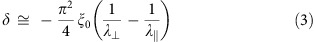


with *ξ*_0_ being the zero-temperature coherence length, which is a measure of the strength of the pairing and *λ*_⊥,||_ being the quasi-particle mean-free path perpendicular or parallel to the strands.

For *T*_c⊥_<*T*<*T*_c||_, this GL theory predicts that the system will be in the polar phase (*A*_⊥,y_=0, *A*_||_>0). For lower temperature, one finds either a distorted A-phase, where *A*_⊥_=0 and *A*_||_>*A*_y_>0 or a distorted B-phase, where *A*_*y*_=0 and *A*_||_>*A*_⊥_>0. The relative stability of these phases depends on the magnitude of the *β* terms in equation (1). Quasi-particle mean-free paths in the nematic aerogel are expected to be relatively long (>450 nm (ref. 19)), compared with the mean-free paths of ordinary silica aerogel (∼100 nm). Thus, we assume that the *β* parameters are only weakly affected by the disorder, and in our analysis, we use the bulk values given in ref. 23.

The degree of polar distortion can be parameterized by *A*_*y*_*/A*_||_ in the A-phase and *A*_⊥_*/A*_||_ in the B-phase. Both these ratios become smaller at lower pressures (corresponding to more polar distortion), and all three phases become less distinct. The A-to-B transition is first order and is hysteretic, while A-to-polar or B-to-polar transition is second order and should be free of hysteresis. Recent theoretical work argues that this Ginzburg–Landau theory may breakdown at high pressures, with several competing possibilities for the high-temperature phase^21^. Our measurements suggest that this breakdown does indeed occur, but they cannot distinguish between the various possible high-temperature phases.

### Evidence for phase transitions in superfluid fraction data

**Figure 3:**
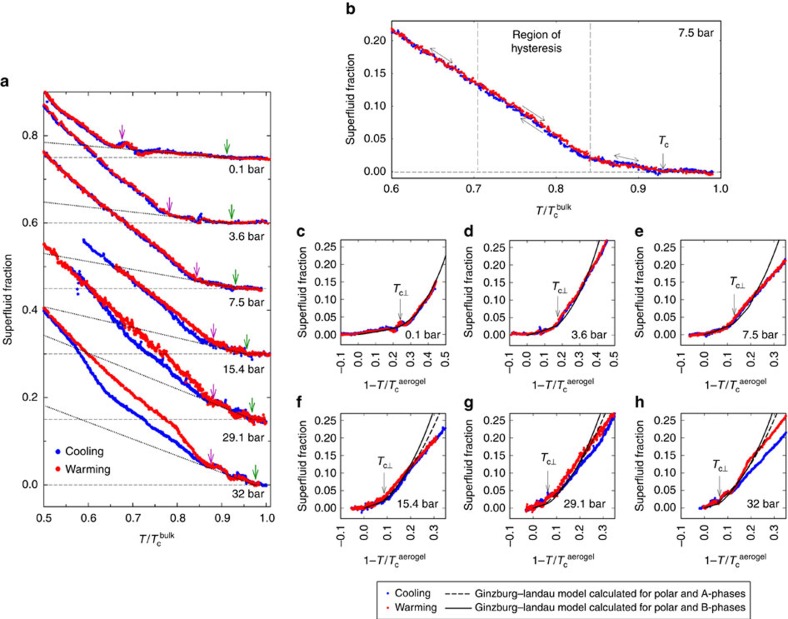
Superfluid fraction data. (**a**) Experimental data for the measured superfluid fraction versus *T*/*T*_c_^bulk^ for both cooling and warming at each of the experimental pressures. Data for each adjacent pressure are offset by 0.15 in the *y* direction for clarity. Dashed lines indicate the zero superfluid fraction level for each pressure. Green arrows mark the superfluid transition temperature for the ^3^He in the aerogel (*T*_c_). Magenta arrows indicate the point at which change in the superfluid fraction data is observed (*T*_kink_). Dotted lines match the data between *T*_c_ and *T*_kink_ and serve as guides to the eye, aiding to identify the precise location of *T*_kink_. (**b**) Data for 7.5 bar. A region of slight hysteresis between cooling and warming is bounded by two vertical dashed lines. (**c**–**h**) Superfluid fraction data (cooling and warming) plotted alongside the superfluid fraction calculated using a Ginzburg–Landau (GL) model for the ^3^He in the nematically ordered aerogel. At high pressures, both A and B phases are present, whereas at low pressures, only B-phase is observed. Data are plotted versus 1−*T/T*_c_, where *T*_c_ is the temperature of the superfluid transition in aerogel. The temperature, *T*_c⊥_, at which the components of the order parameter perpendicular to the strands become nonzero is indicated for each pressure with an arrow.

Our torsional oscillator experiment probes the superfluid density in the direction perpendicular to the pendulum axis, and hence perpendicular to the aerogel strands. Data for the superfluid fraction versus *T/T*_c_^bulk^ for six different pressures ranging from 32 bar, down to 0.1 bar are shown in Fig. 3a. At high pressures (32, 29.1 and 15.4 bar), a clear hysteresis loop is seen between warming and cooling, indicating a first-order phase transition. Guided by the results from NMR experiments done on a similar sample^15^,^16^,^17^ and by the GL model predictions, we identify these as polar-distorted B and polar-distorted A. As pressure is lowered, the hysteresis loop gets less pronounced, indicating a larger degree of polar distortion. Experimental uncertainty in our data is characterized by the relative spread of the data points. In addition, a small systematic error could arise due to the possible thermal lag between our thermometer and the experimental cell. Differences between cooling and warming at 3.6 and 0.1 bar are attributed to this systematic error.

At temperatures slightly above the hysteresis loop, on both cooling and warming, we observe a change in slope in the superfluid fraction versus *T*. The change in slope is especially pronounced at low pressures. We label the temperature of this feature as *T*_kink_. At 7.5 bar (Fig. 3b), data for cooling and warming overlap at low temperatures and near *T*_c_. A difference between the cooling and warming traces larger than the experimental uncertainty is observed at intermediate temperatures. This difference is too large to be due to thermal lag. Instead, we associate the difference with an A-to-B transition. In addition, we observe a very sharp and pronounced kink in the superfluid fraction data. Thus, we conclude that there are three superfluid phases present. Near *T*_c_ a high-temperature phase is stable, which transitions on cooling to a distorted A phase. Cooling through the region of hysteresis the distorted A-phase transitions to a distorted B-phase. On warming, the distorted B-phase persists through the region of hysteresis until it transitions to the high-temperature phase just below *T*_c_. We identify *T*_kink_ as the temperature of the phase transition between a high-temperature superfluid phase and A or B phase. No hysteresis is associated with the transition at *T*_kink_, therefore the transition is second order. The superfluid phase right below *T*_c_ is identified by NMR as an equal spin pairing phase^15^,^16^,^17^ (both the A-phase and polar phase are characterized by equal spin pairing).

## Discussion

To test whether *T*_kink_ is related to *T*_c⊥_, we look for the value of the anisotropy parameter *δ* in equation (1) such that we obtain the best match between the predicted values for *T*_c⊥_ and the observed *T*_kink_. The resulting value for *δ* is of a similar magnitude compared with the one predicted in equation (3), as we insert the values for *λ*_⊥,||_ measured by spin diffusion (450 and 850 nm)^19^. *T*_kink_ conforms to our prediction for *T*_c⊥_ reasonably well at low pressures (0.1, 3.6, 7.5 and 15.4 bar). At high pressures (29.1 and 32 bar), the agreement is not as good and the kink is seen at lower temperatures than the predicted location of *T*_c⊥_. Minimizing the Ginzburg–Landau free energy in equation (1), we obtain the values for the equilibrium order parameter ***A*** and calculate the expected superfluid fraction (Supplementary Note 1). The calculated values, guided by our model, assume a transition from a pure polar to a polar-distorted A or B phase (and not merely a crossover) and a degree of superfluid order parameter suppression due to the proximity of the aerogel strands (Supplementary Note 1; Supplementary Fig. 1). We see very good agreement, especially at low pressures, between the data and calculated values (Fig. 3c). This leads us to conclude that at low pressures, the high-temperature phase has characteristics that correspond to the polar phase, while at high pressures it has additional structure. The system thus displays characteristics of two distinct transitions associated (by G-L theory) with the highly anisotropic mean free paths. The first transition is from the normal to the pure Polar phase and the second to the low temperature A or B phase. These latter phases evolve continuously with diminishing polar distortion as the temperature is lowered. This interpretation is also consistent with NMR measurements, which were done with an aerogel sample that was grown by the same process as ours. They found a larger frequency shift at low pressures than at high pressures^15^,^16^,^17^. Such an evolution of the frequency shift is consistent with a polar-like phase evolving into another structure, but even at the lowest pressures, the frequency shift observed in the NMR measurements was somewhat smaller than what one expects for a pure polar phase. Furthermore, by exposing the superfluid to a rapid series of large NMR pulses, the Moscow group managed to create a spin glass state between the A and B transition and *T*_c_. A spin glass state cannot be created in the polar phase. We note, however, beyond the possibility of aerogel sample differences, the differences of the interpretation between the NMR measurements and this experiment can be explained the following way. If there is a coexistence of a polar and A-phase localized in different parts of the sample, NMR would measure a spatial average that would appear as a highly polar-distorted A-phase. In contrast, torsion pendulum experiments probe the component of the superfluid fraction tensor in the flow direction perpendicular to the aerogel strands. The superfluid density samples the gap along the equatorial nodal line of the Fermi surface leading to the strong superfluid fraction suppression. Even if not all the fluid in the torsion pendulum head is in the polar phase, as long as there is no path connecting the regions of non-polar phase throughout the pendulum head, the superfluid fraction suppression would still be similar to that of the case of having only polar phase in the sample. Torsion pendulum measurements are thus inherently more sensitive to the presence of the polar phase compared with NMR. In addition, the Moscow group recently observed clear evidence for a fully polar phase of superfluid ^3^He embedded in a different type of nematic aerogel using NMR^18^. Both results confirm the prediction that the strong anisotropy of the nematically ordered aerogel matrix promotes the emergence of the polar phase^5^.

**Figure 4:**
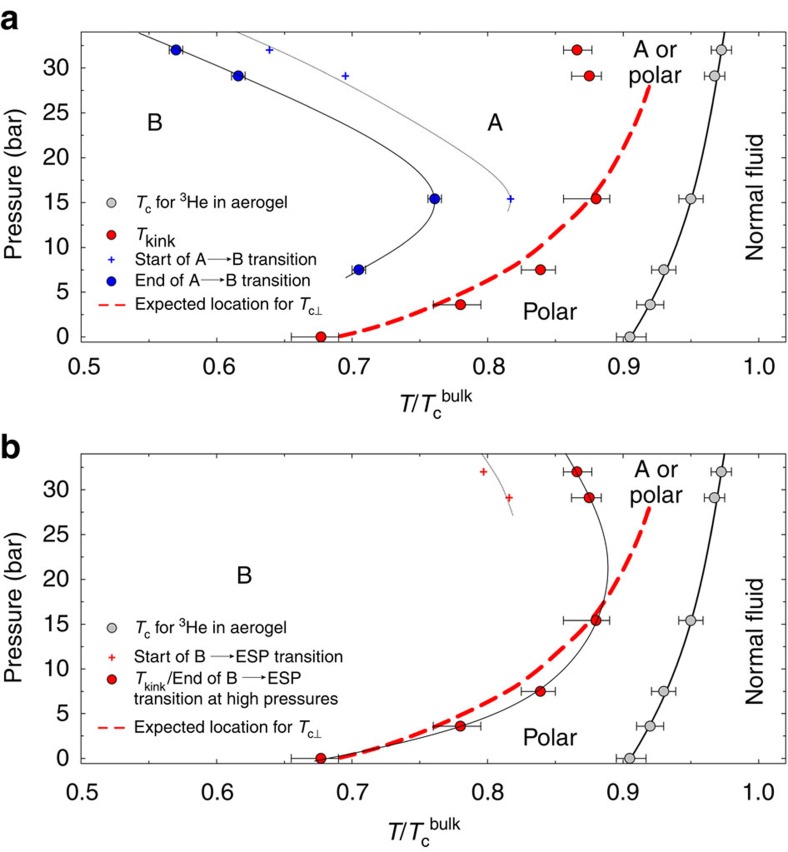
Phase diagram. (**a**) Experimental phase diagram on cooling. (**b**) Experimental phase diagram on warming. Shown are the locations for the experimentally observed phase transitions. Black lines (solid and dotted) connecting the experimental data points are guides to the eye. Due to the experimental noise in our data, there is some uncertainty in locating the precise temperatures at which the superfluid fraction data deviated from the bulk contribution (*T*_c_ for the ^3^He in aerogel), the data changes slope (*T*_kink_) and the locations at which the cooling and warming traces join (end of A-to-B and end of B-to-ESP transitions). These uncertainties are indicated with appropriate error bars.

We summarize the observed phase transitions and our inferences for the natures of the superfluid phases occupying each region of the phase diagram in Fig. 4a,b.

In this article, we have described a series of experiments that reveal striking new phenomena in the superfluid phases of ^3^He embedded in a highly oriented aerogel. Both A and B phases are polar-distorted compared with their bulk counterparts, and their region of stability is markedly different compared with the bulk. We see a high-temperature phase that has no analogue in the bulk. We argue that at low pressures, this superfluid is the polar phase.

## Methods

### Sample preparation and experimental set-up

The aerogel was grown at theLeypunsky Institute of Physics and Power Engineering in Obninsk, Russia, using the same recipe as the aerogel sample used in refs 15, 16, 17, but from a different batch. The aerogel sample had an estimated density of ∼30 g cm^−3^. Its physical properties and method of growth are described in ref. 24. Aerogel was cut into the shape of a cube with sides of ∼5 mm and coated with epoxy. The epoxy used (Tra-bond 2151) had high viscosity and high contact angle with the aerogel surface, which prevented the epoxy from being drawn into the aerogel voids by capillary action. The rest of the torsion pendulum head in which the coated aerogel sample was encapsulated was made from Stycast 1266 epoxy. The torsion rod and the body of the pendulum were made from annealed coin silver (90% silver and 10% copper). Photographs of the torsion pendulum are shown in the Supplementary Fig. 2. We drove and detected the pendulum motion electrostatically, keeping it close to resonance using a digital phase locked loop. The resonant frequency of the mode we excited the pendulum at was ∼476 Hz and the quality factor ranged from 10^5^ to 7 × 10^5^ at mK temperatures. Upon cooling the pendulum below the superfluid transition, the superfluid fraction of the fluid in the aerogel decouples from the pendulum and a decrease in its period is observed. The ratio of the moment of inertia of the fluid in the aerogel sample to the moment of inertia of the rest of the pendulum was about 1 part in 10^4^. We can resolve the period to ∼1 part in 10^7^ giving us ample resolution to resolve the superfluid fraction *ρ*_s_/*ρ*.

### ^4^He coverage

Measurements for 7.5, 15.4, 29.1 and 32 bar were done with pure ^3^He. Because measurements in Moscow were done after the aerogel strands were preplated with 2.5 atomic layers of ^4^He, we also carried out measurements with ^4^He added (∼2 layers). Data for 0.1 and 3.6 bar as well as repeated measurements at 15.4 and 29.1 bar were taken with ^4^He preplating. No substantial change in the transition temperatures at 15.4 and 29.1 bar was seen between measurements with or without preplated ^4^He surfaces. It is not clear if the ^4^He changed the specularity at the aerogel surface. Preplating with ^4^He, however, changed the spectrum of the sound modes present in the fluid (more details on the sound modes can be found in Supplementary Note 2). Sound mode resonances can reveal differences in superfluid textures between the different superfluid states as discussed in Supplementary Note 3 and illustrated by Supplementary Fig. 3.

### Determination of superfluid fraction from the period shift

Because the shape of the aerogel sample is not rotationally symmetric, the fluid in the corners will contribute to the pendulum moment of inertia even in the superfluid state. To account for this and also for the entrainment of the fluid by the strands of aerogel (Kelvin drag), we calibrated the superfluid fraction by filling the sample with pure ^4^He. Because the healing length of ^4^He is ∼0.1 nm, it is expected that the entire sample should be superfluid (aside from 1 to 2 monolayers of disordered solid on the surfaces). Thus, from a measurement of the frequency of the empty cell, of the pendulum above the lambda transition, and at low temperatures well below the superfluid transition temperature for ^4^He, we determined that three quarters of the fluid inertia decoupled.

The bottom part of the aerogel cube was not fully coated with epoxy to allow the fluid to enter the aerogel, leaving a small gap in the pendulum head that would be occupied by bulk fluid. In addition, the aerogel was grown in layers, and in the scanning electron microscope images of our samples, cracks with the sizes of a few microns were observed between some of the layers, with bulk fluid filling those cracks. We estimate that ∼3% of the inferred superfluid fraction came from the bulk fluid, with this contribution to be subtracted out. See Supplementary Note 4 and Supplementary Fig. 4 for more details on the estimated amount of bulk fluid.

The superfluild fraction was determined from the period of the torsion pendulum, *P*(*T*), through the following expression:


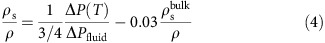


where Δ*P*(*T*) is the difference between the period of the pendulum when all the fluid is fully locked—*P*_full_ and the measured period of the torsion pendulum—*P*(*T*). At *T*_c_, the viscous penetration depth of ^3^He is ∼1 mm, many orders of magnitude larger than the distance between the aerogel strands (∼100 nm), so we can assume that all the fluid is fully coupled with the pendulum. Δ*P*_fluid_ is equal to the difference in the pendulum period between filled, *P*_full_ (*T*), and empty, *P*_empty_. Δ*P*_fluid_ should scale linearly with the density of the fluid. When the sample is pressurized, the axis of the pendulum distorts slightly. To avoid any error due to this, we determine the slope of Δ*P*_fluid_ versus *ρ* through the measured values of (*P*(*T*_c_ at 0.1 bar of ^3^He)—*P*_empty_) and of (*P*(*T*_λ_ at 1 bar of ^4^He)—*P*_empty_) and scale Δ*P*_fluid_ for the other experimental pressures by accounting for the appropriate ^3^He density at that pressure.

### Data availability

The data that support this study are available through Cornell University e-commons data repository at http://hdl.handle.net/1813/44555.

## Additional information

**How to cite this article:** Zhelev, N. *et al*. Observation of a new superfluid phase for ^3^He embedded in nematically ordered aerogel. *Nat. Commun.* **7**, 12975 doi: 10.1038/ncomms12975 (2016).

## Supplementary Material

Supplementary Information Supplementary Figures 1-4, Supplementary Notes 1-4 and Supplementary References

Peer Review File
