# Supplementary material for: Observation of a new superfluid phase for 3He embedded in nematically ordered aerogel
Source: Nat Commun. 2016 Sep 27;7:12975. doi: 10.1038/ncomms12975 (PMC5052659; doi:10.1038/ncomms12975)
Supplement: Supplementary Information — Supplementary Figures 1-4, Supplementary Notes 1-4 and Supplementary References [file ncomms12975-s1.pdf]

Supplementary Figures:

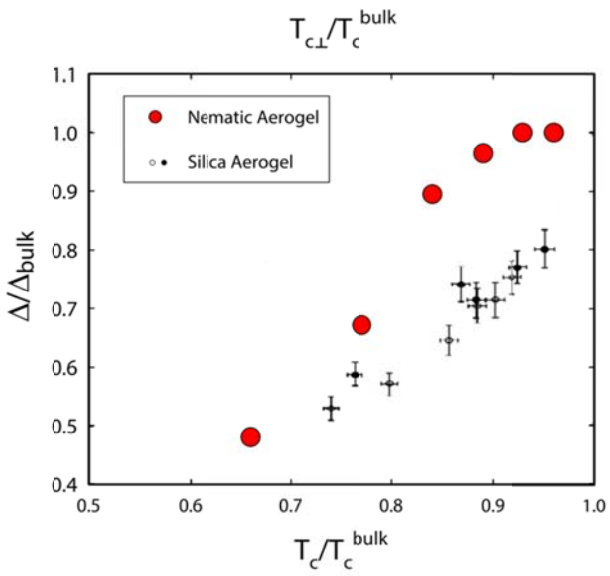

Supplementary Figure 1: Amount of superfluid gap suppression due to aerogel

The ratio of the superfluid gap for the fluid in the aerogel over the superfluid gap of the bulk fluid is plotted versus the superfluid transition temperature suppression for different aerogel samples. Black filled and empty circles represent data from Ref. 1 plotted against T_c/T_c^{bulk} for two isotropic silica aerogel samples with different porosities (99.5% and 98%). Filled red dots indicate the factors by which we scaled the superfluid gap in our GL model calculated superfluid fractions for the present experiment plotted versus $T_{c\perp}/T_c^{bulk}$.

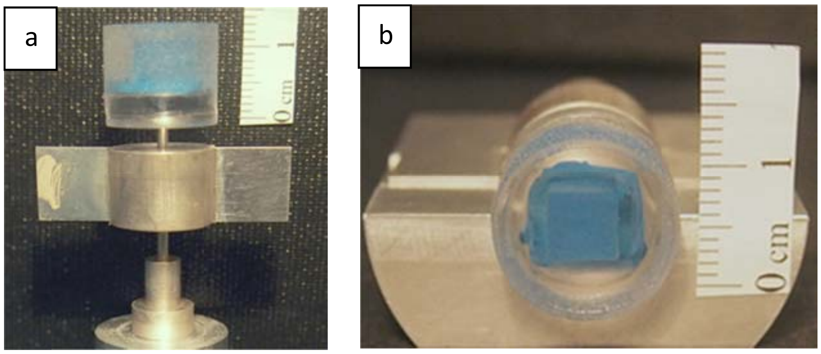

Supplementary Figure 2: Photographs of the torsion pendulum

Photographs of the torsion pendulum after it was dismounted from the cryostat at the end of the experiment. The blue colored cube within the otherwise transparent torsion head is the epoxy coated aerogel sample.

Panel a shows a side view of the pendulum.

Panel b shows a top view.

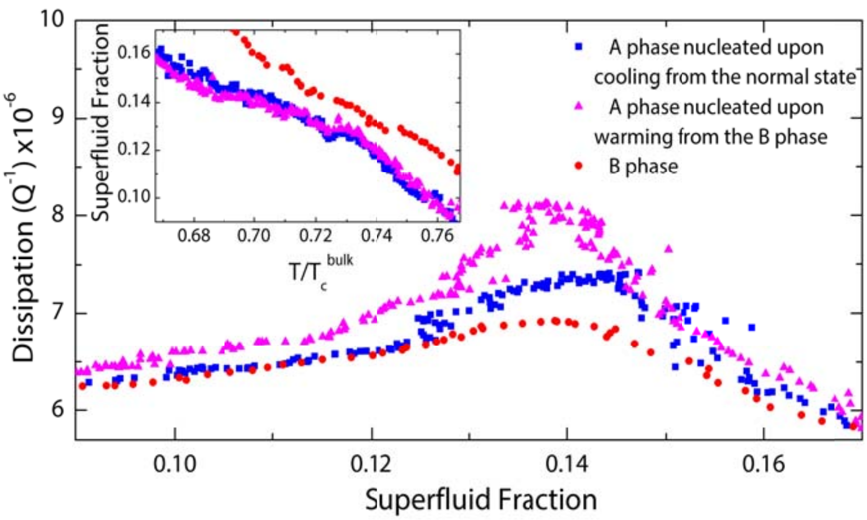

Supplementary Figure 3: Superfluid textural differences revealed by sound resonances

Dissipation peak due to the excitation of a slow-mode sound resonance in the fluid embedded in the aerogel. Inset: Plot for the superfluid fraction versus T/T_c^{bulk} for the values of superfluid fraction/temperature at which the slow-mode sound resonance occurs.

.

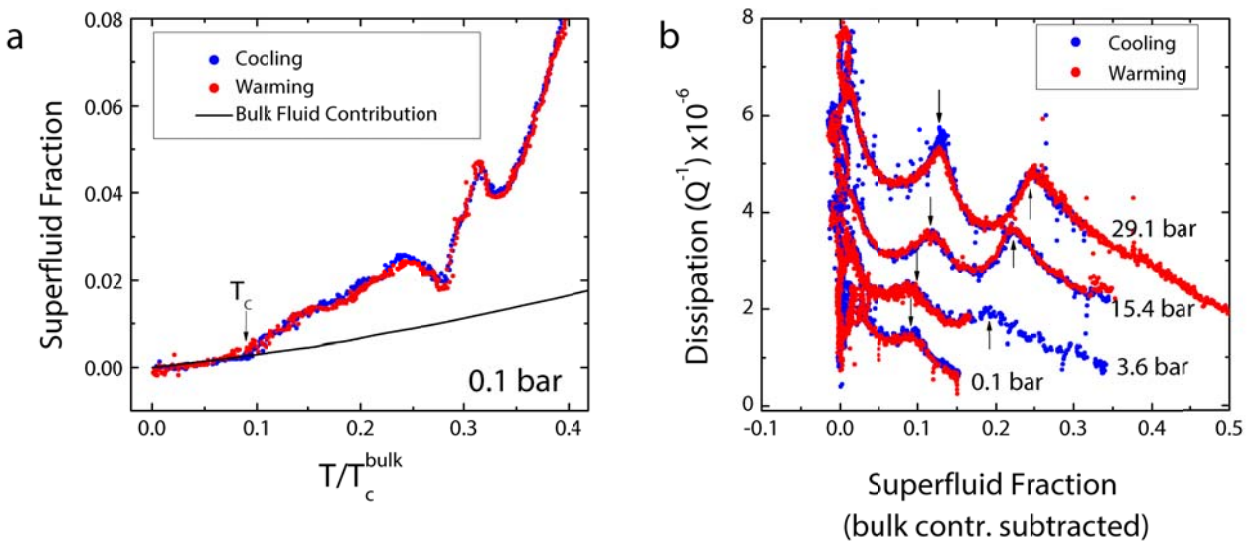

Supplementary Figure 4: Determination of bulk fluid contribution

a. Superfluid fraction versus T/T_c^{bulk} for 0.1 bar. Shown is data on cooling and warming (blue, red dots), as well as the estimated 3% bulk fluid contribution to the superfluid fraction (black line). The superfluid transition for the fluid in the aerogel is marked by an arrow.

b. Dissipation versus superfluid fraction for the fluid embedded in the aerogel (3% bulk contribution is subtracted) at 0.1, 3.6, 15.4, and 29.1 bar. Visible are several sound resonance peaks. We highlight two sets of slow-mode sound resonances, at values of about 0.1-0.12 and 0.2-0.24 for the superfluid fraction. Arrows indicated the expected locations of the dissipation peaks based on the data for 29.1 bar and the ratios of the fluid densities at the other pressures.

Supplementary Note 1: Calculating the superfluid fraction from the GL-model

Having determined the values of the matrix order parameter that minimizes the GL free energy, we calculate the superfluid fraction in the plane of the torsion pendulum head starting from [2]:

$$\rho^0_{n\,ij} = 3\rho\langle \hat{\boldsymbol{k}}_i \hat{\boldsymbol{k}}_j Y_0(\hat{k}, T)\rangle_{\hat{k}} \qquad \text{(Supplementary Equation 1)}$$

$$\boldsymbol{\rho}_\mathrm{n} = \frac{m^*}{m}\left(\mathbf{1} + \frac{1}{3}F^s_1 \frac{\boldsymbol{\rho}^0_\mathrm{n}}{\rho}\right)^{-1} \boldsymbol{\rho}^0_\mathrm{n} \qquad \text{(Supplementary Equation 2)}$$

$$\rho_{s\,\text{in plane}} = \rho - \frac{\rho_{n\,xx} + \rho_{n\,yy}}{2} \qquad \text{(Supplementary Equation 3)}$$

where $\boldsymbol{\rho}_\mathrm{n}$ and $\boldsymbol{\rho}^0_\mathrm{n}$ are 3x3 matrices with components $\rho_{n\,ij}$ and $\rho^0_{n\,ij}$ respectively, m^* is the ^{3}He quasiparticles renormalized mass, and F^s_1 is the Landau parameter. $Y_0(\hat{\boldsymbol{k}}, T)$ is the Yosida function, which is related to the energy density distribution along the Fermi sphere, f:

$$Y_0(\hat{\boldsymbol{k}}, T) = -\int_{-\infty}^{\infty} d\epsilon_{\hat{\boldsymbol{k}}} \left(\frac{\partial f}{\partial E_{\hat{\boldsymbol{k}}}}\right) \qquad \text{(Supplementary Equation 4)}$$

with the information for the gap structure contained in $\partial f/\partial E_{\hat{\boldsymbol{k}}}$.

The presence of impurity scattering in aerogels leads to a reduction of the superfluid gap, as well as the superfluid temperature compared to the bulk [1, 3]. Supplementary Figure 1 shows data compiled in Ref. 1 for the measured superfluid gap over the superfluid gap in the bulk fluid plotted versus the ratio of T_c in aerogel and in bulk for several isotropic (silica) aerogel samples. It is observed that a strong relationship between T_c suppression and gap suppression exists. In our aerogel sample (nematic aerogel), we take $T_{c\perp}/T_c^{bulk}$ to be the relevant parameter that should determine the ratio of the superfluid gap for ^{3}He in the aerogel compared to that in the bulk. We scale the superfluid fractions from Supplementary Equation 3 by the square of the

ratio between the superfluid gap for the fluid within the aerogel and the bulk superfluid gap (superfluid density is proportional to the square of the gap). The ratio that best fits the experimental data is plotted as filled red circles in Supplementary Figure 1 for each of the experimental pressures. These were chosen to be temperature independent, but varied with pressure. As seen in the figure, the factors by which the gap is suppressed that best match the data vary roughly linearly with superfluid transition temperature suppression in a similar fashion as previously observed for the fluid in isotropic silica aerogels.

Supplementary Note 2: Slow-mode sound resonances

When the torsion pendulum frequency crosses the resonant frequency of a standing sound wave mode, these sound resonances appear as dips in the amplitude of the pendulum for the same value of drive, thus they are seen as peaks in the measured dissipation (Q^{-1}). Our procedure for determining the resonant frequency of the pendulum (digital phase locked loop) relies on a Lorentzian resonance response. The presence of sound modes in the "head" of the torsional pendulum results in a distorted complex response which appears as a non-monotonic calculated resonance frequency, and hence the inferred superfluid fraction. The resonances are sufficiently far spaced that we can none-the-less reliably produce a phase diagram.

Starting from the two fluid model for superfluids, there are many ways in which sound can propagate though the liquid medium. In bulk ^{3}He one finds first sound (normal and superfluid components move in-phase), second (normal and superfluid components move out-of-phase) and fourth sound (normal component is clamped, but the superfluid component oscillates) [4]. In aerogel, the normal fluid is well clamped to the aerogel strands, while the superfluid is free to move, as in the case of fourth sound. However, the aerogel is not perfectly rigid, but can

flex as well. Thus sound modes for ^{3}He in aerogel are composite modes in which the aerogel, the normal and the superfluid components all move. Two such modes are possible, the fast mode in which all components move in phase, and a slow mode in which the superfluid component is out-of-phase with the flexing of the aerogel strands and the normal component [5]. The sound velocity of the slow mode, c_s, is related to the speed of sound in aerogel, c_a, and the superfluid fraction, ρ_s/ρ, through the following expression:

$$\frac{c_s}{c_a} = \left(\frac{\rho_a}{\rho(P)}\frac{\rho_s}{\rho}\right)^{1/2} \qquad \text{(Supplementary Equation 5)}$$

where $\rho(P)$ is the density of the fluid at the particular pressure. Since c_s depends on ρ_s/ρ, the slow-mode sound velocity (and therefore the wavelength at a particular excitation frequency) changes rapidly with temperature below the superfluid transition, starting at zero at T_c.

Supplementary Note 3: Superfluid textural differences revealed from sound resonances

While normally a nuisance, the sound resonance dissipation peaks give us some insight into the superflud state. For example, Supplementary Figure 3 shows the dissipation peak for one of the slow-mode sound resonances that is excited at a superfluid fraction of ~ 0.14. We observe that dissipation peak in the A phase is larger than the dissipation peak in the B phase. Furthermore, there is a noticeable difference between the dissipation peak seen in the A phase nucleated upon cooling from the normal state and the A phase nucleated upon warming from the B phase. Such a difference is consistent with the results from Ref. 6, in which A phase was identified to have a "glassy" structure – on a short length scale, the nodes in the Fermi surfaces are aligned, but on a long length scale they are randomly disordered. In Ref. 7, the A phase has different NMR frequency shift depending on whether it was nucleated from the normal state or

from the B phase, with the interpretation being that there is a different degree of long length scale disorder in each of the A phases. In our experiment, we do not see a difference in the measured superfluid fraction (Supplementary Figure 3 inset), but the difference in the sound resonance response suggests that the superfluid texture in the A phase is different in the two cases.

Supplementary Note 4: Determination of the fraction of bulk fluid

The resonant frequency and the quality factor of the oscillator changes below T_c^{bulk}, indicating the decoupling of the superfluid fraction of bulk fluid. A faster rise in the resonant frequency (superfluid fraction) at a lower temperature signals the superfluid transition in the aerogel T_c (Supplementary Figure 4a). Using the values for the bulk superfluid fraction from Ref. 8., and scaling them by a factor of 0.03, we obtain a good fit to the data between T_c^{bulk} and T_c, indicating that the bulk fluid moment of inertia amounts to about 3% of the total fluid inertia in the pendulum.

We can use the slow-mode sound resonance to verify this 3% bulk contribution to the moment of inertia. In particular, we track the slow-mode sound resonance dissipation peaks for each experimental pressure (Supplementary Figure 4b). Sound resonances occur at the same values for c_s for each pressure, and since ρ_a and c_a are fixed, then ρ_s/ρ at the dissipation peaks should scale as the density ρ for different pressures (Supplementary Equation 5). Two sets of arrows in Supplementary Figure 4b indicate the predicted ρ_s/ρ for two of the prominent dissipation peaks in our data. The values ρ_s/ρ for 29.1 bar were set by the data, and the values for ρ_s/ρ at the lower pressures were determined by multiplying ρ_s/ρ (29.1 bar) by

$\rho(P)/\rho(29.1\ \mathrm{bar})$. We observe good agreement between the observed and predicted location of the sound resonance dissipation peaks, which would not have been the case if we had incorrectly estimated the bulk contribution.

Supplementary References:

1. Lawes G. & Parpia J. M., Estimate of the gap parameter for superfluid ^{3}He in aerogel. *Phys. Rev. B* **65**, 092511 (2002).

2. Vollhardt, D., Wölfle, P., *The Superfluid Phases of Helium 3*, Dover Publications, Mineola, New York (2013).

3. Thuneberg, E. V., Yip, S. K., Fogelström, M. & Sauls, J. A., Models for Superfluid ^{3}He in Aerogel. *Phys. Rev. Lett.* **80**, 2861 (1998).

4. Leggett, A. J., A theoretical description of the new phases of liquid ^{3}He. *Rev. Mod. Phys*. **47**, 331 (1975).

5. Golov, A.,Geller, D. A., & Parpia, J. M., Acoustic Spectroscopy of Superluid ^{3}He in aerogel. *Phys. Rev. Lett.* **82**, 3492 (1999).

6. Askhadullin, R. S., *et al.*, Phase Diagram of superfluid ^{3}He in “nematically ordered” aerogel. *JETP Lett.* **95** (6), 355-360 (2012).

7. Askhadullin, R. S., *et al.*,Anisotropic 2D Larkin-Imry-Ma state in polar distorted ABM phase of ^{3}He in “nematically ordered” aerogel. *JETP Lett.* **100** (10), 747-753 (2014).

8. Parpia, J. M., Wildes, D. G., Saunders, J., Zeise, E. K., Reppy, J. D., & Richardson, R. C., The Temperature and Pressure Dependence of the Normal Fraction of Superfluid ^{3}He-B. *J. Low. Temp. Phys.* **61**, 337-361 (1985).
