# Supplementary material for: Observation of a new superfluid phase for 3He embedded in nematically ordered aerogel
Source: Nat Commun. 2016 Sep 27;7:12975. doi: 10.1038/ncomms12975 (PMC5052659; doi:10.1038/ncomms12975)
Supplement: Peer Review File [file ncomms12975-s2.pdf]

Editorial Note: This manuscript has been previously reviewed at another journal that is not operating a transparent peer review scheme. This document only contains reviewer comments and rebuttal letters for versions considered at Nature Communications.
